# Emergence of Group B *Streptococcus* Disease in Pigs and Porcupines, Italy

**DOI:** 10.3201/eid3006.231322

**Published:** 2024-06

**Authors:** Chiara Anna Garbarino, Simone Bariselli, Giovanni Pupillo, Patrizia Bassi, Andrea Luppi, Roberta Taddei, Alessandro Reggiani, Elisa Massella, Matteo Ricchi, Elena Carra, Ruth N. Zadoks

**Affiliations:** Istituto Zooprofilattico Sperimentale della Lombardia e dell’Emilia Romagna, Brescia, Italy (C.A. Garbarino, S. Bariselli, G. Pupillo, P. Bassi, A. Luppi, R. Taddei, A. Reggiani, E. Massella, M. Ricchi, E. Carra);; University of Sydney Faculty of Science, Sydney School of Veterinary Science, Camden, New South Wales, Australia (R.N. Zadoks)

**Keywords:** group B *Streptococcus*, *Streptococcus agalactiae*, pigs, wildlife, porcupines, foodborne, food safety, respiratory infections, bacteria, Italy, antimicrobial resistance, zoonoses

## Abstract

We describe group B *Streptococcus* linked to disease in farmed pigs and wild porcupines in Italy. Occurrence in pigs was attributed to transmission from nonpasteurized bovine milk whey. Antimicrobial-resistance profiles in isolates from porcupines suggest no common source of infection. Our findings expand the known host range for group B *Streptococcus* disease.

*Streptococcus agalactiae* (group B *Streptococcus* [GBS]) is a major pathogen of humans, cattle, aquatic species, and camels (*1**­*–*4*). GBS has been detected in pork but has not been associated with disease in pigs (*5*). Transmission between humans and animals may occur in multiple directions, and the organism’s genome plasticity enables it to acquire accessory genome content that confers survival advantages in new niches, facilitating adaptation and onward transmission within new host species (*6*–*8*). We describe emergence of GBS as a cause of disease in domestic pigs (*Sus scrofa domesticus*) and wild porcupines (*Hystrix cristata*) in Italy.

## The Study

**Figure 1:**
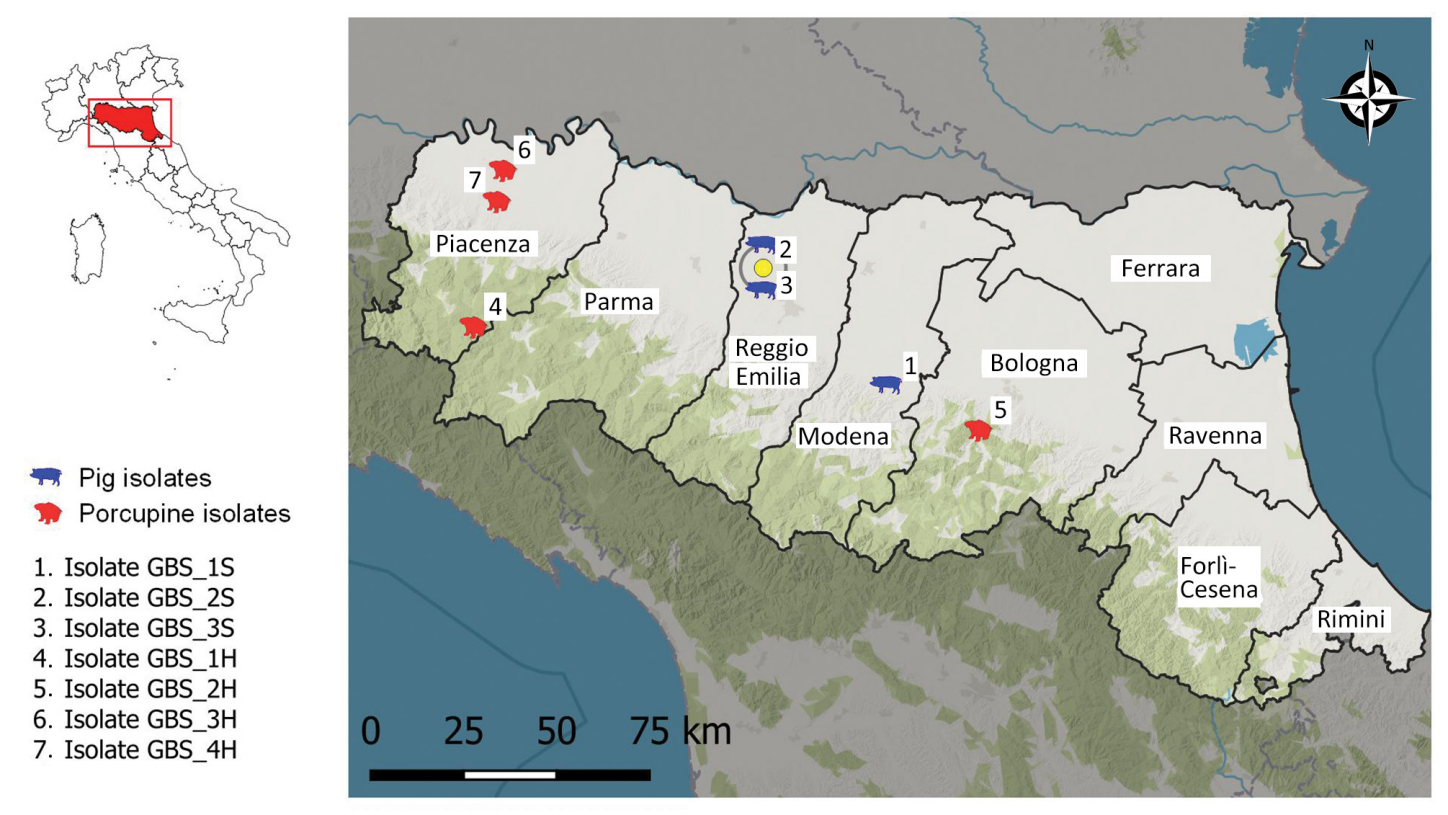
Geographic origin of group B *Streptococcus* bacterial isolates from pigs (*Sus scrofa*) and porcupines (*Hystrix cristata*) in Emilia Romagna region, northern Italy. Numbers indicate bacterial isolate for each diagnostic submission based on host and sequential number: GBS, group B *Streptococcus*; H, *H. cristata*; S, *S. scrofa*. Inset shows location of the region

In 2022, GBS was isolated during disease investigations on pig fattening farms in the provinces of Modena (farm 1, closed farming system with high biosecurity standards) and Reggio Emilia (farm 2, open farming system with low biosecurity standards), Emilia Romagna region, northern Italy (Figure 1; Appendix Table 1). The affected farms were ≈50 km apart and had no known links (e.g., through animals, feed, veterinarians, or workers). Neither farm had direct contact with dairy farms. Both farms used bovine milk whey as a feed ingredient in their pig fattening units. Farm 1 obtained whey from a single milk processing company, and farm 2 used multiple suppliers. Whey was used within 24 hours of receipt but was not heat treated at any stage.

**Figure 2:**
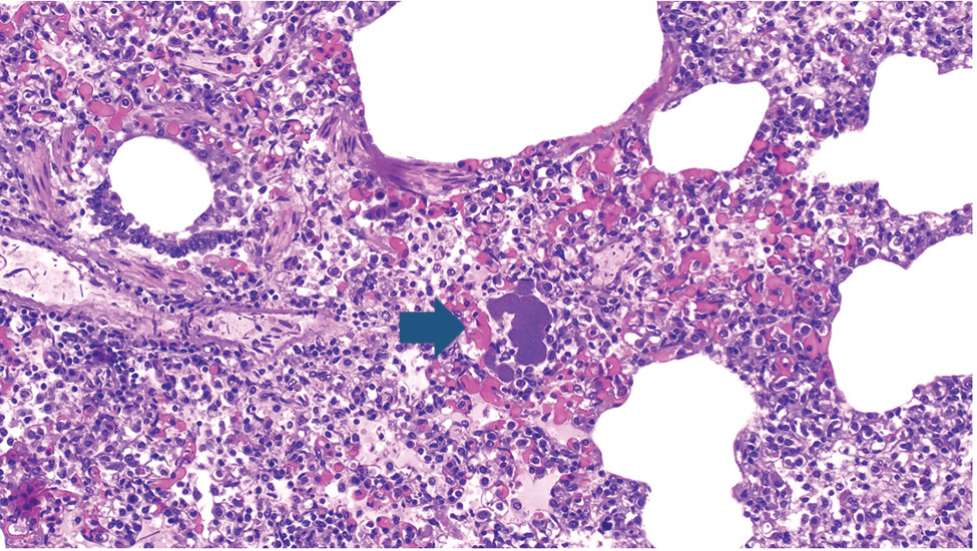
Bacterial embolus (arrow) caused by group B *Streptococcus* infection in a lung section of a pig (*Sus scrofa*) from a pig farm in the Modena Province, northern Italy. Group B *Streptococcus* was detected in the kidney, liver, and heart, indicating disseminated infection caused by septicemia. Hematoxylin–eosin staining; original magnification ×10.

In March 2022, farm 1 submitted the carcass of a pig found dead after 2 days of depression and anorexia to the Istituto Zooprofilattico Sperimentale della Lombardia e dell’Emilia Romagna (IZSLER; Brescia, Italy); no other animals in the group showed any signs of disease. On examination, we found lesions mainly in the lungs, liver, and heart, and histologic examination showed bacterial emboli containing GBS in lymphatic and pulmonary tissue (Figure 2). Farm 2 submitted samples from 1 pig in July 2022 and from 3 other pigs in December 2022, all having respiratory symptoms (coughs and dyspnea). On examination, we observed interstitial edema and multiple stages of pleuritis in the lungs and purulent catarrhal bronchopneumonia and mild fibrinous pericarditis in the pigs submitted in December. We isolated GBS from the lungs and lower airways of pigs from each submission (Appendix Table 1).

IZSLER also receives wildlife specimens from the Emilia Romagna region, where a regional wildlife surveillance plan has been in force since 2017, covering wild animals found dead or animal samples or carcasses from wildlife rescue centers. The plan covers numerous species, including porcupines, which are found in flat and hilly areas of the Emilia Romagna region and in many other regions of Italy. Porcupines mostly live in woods and areas with caves and bushes, but human interaction on farms or in gardens is possible. At the beginning of March 2023, a porcupine from San Gregorio of Ferriere, a Piacenza Province municipality located in the Apennine Mountains, was admitted to the Piacenza Rescue Centre (CRAS PC) with severe respiratory signs. CRAS PC collected a tracheal swab sample upon admission and submitted it to IZSLER with a request for bacteriologic culture and antimicrobial susceptibility testing to guide the animal’s treatment. GBS was isolated in pure culture from the tracheal swab sample. The porcupine died within days, and the carcass was not submitted for necropsy. In March 2023, a young female porcupine was found dead in Sasso Marconi municipality, Bologna Province, and submitted to IZSLER. Necroscopic examination revealed numerous abscesses in the right lung and 1 inside the thoracic cavity. GBS was isolated from the abscesses in pure culture. In May and July 2023, two adult male porcupines were found in Piacenza Province, the first in Pittolo, a lowland municipality, and the second in Rivergaro. Both were submitted to CRAS PC and then to IZSLER. According to CRAS PC, the first porcupine was in poor condition and was euthanized at the rescue center; the second porcupine was found deceased. In both adult male porcupines, our necroscopic and bacteriologic analysis revealed hematomas and injuries consistent with multiple traumas, lung impairment with increased consistency and diffuse congestion, and the presence of GBS in pure culture in the lungs (Appendix Table 1).

We used the Sensititer (Thermo Fisher Scientific, https://www.thermofisher.com) for antimicrobial susceptibility testing of 1 isolate per diagnostic submission (Appendix Table 1) following Clinical and Laboratory Standards Institute guidelines (*9*). In the absence of specific breakpoints for GBS in pigs and porcupines, we used breakpoint values for *Streptococcus* in pigs (Appendix Table 2). The isolates from each of the 3 porcine submissions demonstrated resistance to erythromycin, tetracycline, and kanamycin (high level), whereas the 4 porcupine isolates were susceptible to most compounds, including kanamycin (Appendix Table 2). According to 7-gene multilocus sequence typing, the GBS isolates from each of the 7 diagnostic submissions belonged to sequence type (ST) 103 (*10*).

## Conclusions

Detection of GBS in the tongue, tonsils, or intestines from pigs at slaughter has been reported previously without evidence of pathology (*5*,*11*). In this article, we describe GBS as a primary pathogen in pigs and porcupines on the basis of antemortem or postmortem evidence of respiratory disease because the GBS bacterial pathogen was isolated in pure culture from lung lesions and because the clinical and pathologic manifestations were consistent with GBS respiratory infection in humans, camels, and rabbits (*4**,**12*). For the porcupines, we speculate respiratory disease caused by GBS led to submission to the wildlife center either directly (sick porcupine) or indirectly, after sick animals were injured by traffic, which would explain the observed multiple traumas.

Human-to-animal transmission is possible for GBS (*3*,*7*). Such transmission seems unlikely in this case because the porcupines were positive for GBS before contact with the rescue centers. ST103 has also not been detected in the human population in the Emilia Romagna region (*13*). Introduction of GBS to the pig farms from raw milk whey is possible because ST103 is known to affect dairy herds in the Emilia Romagna region (*13*). Foodborne transmission of GBS has been documented previously (*6*,*14*). Source farms for the whey were not traced, but tracing could be attempted in future cases. The route of transmission to porcupines is unknown. Transmission from cattle to porcupines cannot be ruled out, possibly through dissemination of ST103 in bovine feces into the environment (*15*). Bovine ST103 isolates from the region, like the pig isolates in this study, are tetracycline-resistant and high level kanamycin-resistant (*13*). Isolates from the porcupines were fully susceptible, however, suggesting that an independent population of GBS might be present in the porcupine population.

Although a common exposure route was not identified and antimicrobial resistance profiles differed between GBS isolates from the 2 host species, all necropsied pigs and porcupines were infected with ST103. In Europe, ST103 has primarily been found in cattle, where it may have an environmental transmission cycle, in contrast to most other GBS strains that cause bovine mastitis (*7*,*15*). A single-locus variant of ST103 (ST651) was also the most common sequence type found in pig organs in Hong Kong (*10*). Our findings raise concerns about the ability of GBS ST103 and closely related sequence types to adapt to multiple host species and organs systems and highlights risks for future emergence in additional host species.

Appendix Additional information about emergence of group B *Streptococcus* disease in pigs and porcupines, Italy.
