# Supplementary material for: Emergence of Group B Streptococcus Disease in Pigs and Porcupines, Italy
Source: Emerg Infect Dis. 2024 Jun;30(6):1228–31. doi: 10.3201/eid3006.231322 (PMC11138975; doi:10.3201/eid3006.231322)
Supplement: Appendix — Additional information about emergence of group B Streptococcus disease in pigs and porcupines, Italy. [file 23-1322-Techapp-s1.pdf]

Article DOI: https://doi.org/10.3201/eid3006.231322

EID cannot ensure accessibility for supplementary materials supplied by authors. Readers who have difficulty accessing supplementary content should contact the authors for assistance.

Emergence of Group B *Streptococcus* Disease in Pigs and Porcupines, Italy

Appendix

Appendix Table 1. Origin (host, location) and nature of lesions in pigs (Sus scrofa, S) and porcupines (Hystrix cristata, H) testing positive for group B Streptococcus (GBS) in Northern Italy. Isolate numbering is based on host species and submission ID.

Host species	Submission ID	Sample type	Date	Province	Signs	Macroscopic and Histology findings	Bacteriological isolate
Pig	1	Carcass	24/03/2022	Castelvetro (Modena) Pre-hilly area Elevation 130 m Farm 1	Two days of depression and anorexia	Liver: small, nodular, whitish lesions in the left hepatic lobe; Heart: mild siero-fibrinous pericarditis, tricuspid valve endocarditis and nodular lesions in the right atrial wall; Lung: pulmonary interstitial edema, firm pleural adhesions and n. 2 abscesses in left pulmonary lobe Histology: moderate interstitial pneumonia, severe subacute fibrino-suppurative endocarditis, mild myocarditis, moderate chronic pericarditis and moderate lymphoid hyperplasia in lymph node. Multiple septic emboli in pulmonary and cardiac tissue	*S. agalactiae* (Isolated from lung, kidney, liver and heart) Isolate GBS 1S
	2	Lung Heart Kidney Spleen	12/07/2022	Campegine (Reggio Emilia) Plain area Elevation 35 m Farm 2	Respiratory signs: generalized coughs and dyspnoea	Lung: pulmonary interstitial edema and mild diffuse fibrinous pleuritis	*S. agalactiae* (in lungs and lower airways) Isolate GBS 2S
	3	Lung Heart Kidney Spleen	05/12/2022	Campegine (Reggio Emilia) Plain area Elevation 35 m Farm 2	Respiratory signs: generalized coughs and dyspnoea	Lungs: pulmonary interstitial edema, different stages of pleuritis, purulent catarrhal bronchopneumonia Heart: mild fibrinous pericarditis.	*S. agalactiae* (in lungs and lower airways) Isolate GBS 3S
Porcupine	1	Tracheal swab performed when alive	03/03/2023	San Gregorio of Ferriere (Piacenza) Elevation 626 m	Respiratory signs. Sudden death at Rescue Center	N/A	*S. agalactiae* Isolate GBS 1H
	2	Carcass (young female)	18/03/2023	Sasso Marconi (Bologna) Municipality Elevation 128 m	Found dead with extensive cutaneous eschar of the back of the head and back in the lumbar region	Extensive cutaneous eschar of the back of the head and back in the lumbar region; numerous abscesses were observed in the right lung and one in the thoracic cavity.	*S. agalactiae* (From lung abscesses) Isolate GBS 2H
	3	Carcass (adult male)	28/05/2023	Pittolo (Piacenza) lowland municipality	Hematomas and injuries referable to multiple trauma. Fracture of the pelvis. Euthanized at rescue center	Lung congestion, increased parenchyma consistency	*S. agalactiae* (From lung, not found in other organs) Isolate GBS 3H
	4	Carcass (adult male)	03/07/2023	Loc. Trebbiola of Rivergaro (Piacenza) Elevation 128 m	Found dead	Lung congestion, increased parenchyma consistency	*S. agalactiae* (From lung, not found in other organs) Isolate GBS 4H

Table 2. MICs of antimicrobial compounds against Group B *Streptococcus* (GBS) isolates from pigs (*Sus scrofa domesticus*, S) and porcupines (*Hystrix cristata*, H). Each isolate (S for pig isolates, H for porcupine isolates) represents an epidemiologically independent diagnostic submission (indicated by a number which corresponds to the map in Figure 1). S = susceptible, I = intermediate, R = resistant based on GBS breakpoints in agreement with CLSI VET08 4th edition, CLSI M100 29th edition, and EUCAST v.11.0.

	Breakpoint*			Isolate GBS 1S		Isolate GBS 2S		Isolate GBS 3S		Isolate GBS 1H		Isolate GBS 2H		Isolate GBS 3H		Isolate GBS 4H	
Antimicrobial	S	I	R														
Amoxicillin + clavulanic acid	≤0,25/0,12	0,5/0,25	>1/0,5	≤0,25	S	≤0,25	S	≤0,25	S	≤0,25	S	≤0,25	S	≤0,25	S	≤0,25	S
Ampicillin	≤0,5	1	≥2	0.12	S	0.12	S	0.12	S	0.12	S	0.06	S	0.12	S	0.12	S
Cefazolin	≤2	4	≥8	≤0,25	S	≤0,25	S	≤0,25	S	≤0,25	S	≤0,25	S	0.5	S	≤0,25	S
Ceftiofur	≤2	4	≥8	≤0,25	S	≤0,25	S	≤0,25	S	≤0,25	S	≤0,25	S	≤0,25	S	≤0,25	S
Enrofloxacin	≤0,5	1	≥2	≤0,25	S	1	I	1	I	1	I	1	I	1	I	1	I
Erythromycin	≤0,25	0.5	≥1	>8	R	>8	R	>8	R	0.06	S	≤0,03	S	0.06	S	0.06	S
Florfenicol	≤2	4	≥8	≤2	S	≤2	S	≤2	S	≤2	S	≤2	S	≤2	S	≤2	S
Kanamycin (High level)	≤250	-	>500	>500	R	>500	R	>500	R	≤250	S	≤250	S	≤250	S	≤250	S
Oxacillin	≤2	-	>4	0.5	S	0.5	S	0.5	S	0.5	S	0.5	S	0.5	S	0.5	S
Penicillin	≤0,25	0.5	≥1	0.06	S	0.06	S	0.06	S	0.12	S	0.06	S	0.12	S	0.06	S
Rifampin	≤0,06	-	>0,5	0.25	I	0.25	I	0.25	I	0.25	I	≤0,06	S	0.5	I	0.25	I
Tetracycline	≤2	4	≥8	>16	R	>16	R	>16	R	0.5	S	≤0,25	S	≤0,25	S	0.5	S
Trimethoprim + sulfamethoxazole	≤2/38	4/76–8/152	≥16/304	≤0,12	S	≤0,12	S	≤0,12	S	≤0,12	S	0.25	S	≤0,12	S	≤0,12	S

*S, Sensitive; I, Intermediate; R, Resistant
